# Lectins bring benefits to bones

**DOI:** 10.7554/eLife.22926

**Published:** 2016-12-13

**Authors:** Charles KF Chan, Ryan C Ransom, Michael T Longaker

**Affiliations:** 1 Hagey Laboratory for Pediatric Regenerative Medicine and Department of Surgery, the Departments of Pathology and Developmental Biology and the Institute for Stem Cell Biology and Regenerative Medicine, Stanford University, Palo Alto, United States; 2 Hagey Laboratory for Pediatric Regenerative Medicine and Department of Surgery and the Institute for Stem Cell Biology and Regenerative Medicine, Stanford University, Palo Alto, United States; 3 Hagey Laboratory for Pediatric Regenerative Medicine and Department of Surgery and the Institute for Stem Cell Biology and Regenerative Medicine, Stanford University, Palo Alto, United Stateslongaker@stanford.edu

**Keywords:** osteogenesis, skeletal stem cells, osteoporosis, Mouse

## Abstract

The discovery that proteins called c-type lectins promote bone growth could lead to new treatments for age-related bone disorders.

**Related research article** Yue R, Shen B, Morrison SJ. 2016. Clec11a/osteolectin is an osteogenic growth factor that promotes the maintenance of the adult skeleton. *eLife* **5**:e18782. doi: 10.7554/eLife.18782

Lectins are a diverse group of proteins that are attracted to carbohydrates and biomolecules that contain carbohydrate groups, including varieties of sugars and sugar-modified proteins and lipids. This attraction, which is highly specific, allows lectins to influence a wide range of biological processes. For example, many bacterial and mammalian lectins control how cells move and adhere to each other (Ghazarian et al., 2011), while some plant lectins, notably ricin and abrin, are renowned for their lethality (Olsnes, 2004).

A widely studied group of mammalian lectins are the c-type lectins, which are so named because they require a calcium ion to work correctly. Examples of c-type lectins include thrombomodulin, which regulates platelet-dependent coagulation, and selectins, which control the movement of white blood cells during inflammation (Cummings et al., 2009). Now, in eLife, Rui Yue, Bo Shen and Sean Morrison at the University of Texas Southwestern Medical Center report that Clec11a, a type of c-lectin that is expressed at high levels by cells in bone marrow, stimulates bone regeneration (Yue et al., 2016).

Since other types of lectins are known to help white blood cells interact with other cells, Yue et al. initially expected that Clec11a might regulate how new blood cells form in the bone marrow. To test this hypothesis, they generated knockout mice that lacked Clec11a. However, the effects of this deficiency were unexpectedly mild. The knockout mice developed normally and young adults were not significantly anatomically different from wild-type mice. The knockouts also had normal levels of hematopoietic stem cells, and these developed into mature blood cells at a normal rate.

About the only detectable difference exhibited by the knockout mice was a reduction in overall bone volume. This deficit increased over time and by 16 months of age, knockout mice had just over half the volume of bone of the wild-type mice. Further analysis revealed that the bone-mineral density of the knockout mice was also normal. However, significant age-related thinning of cortical bone (the hard outer layer along long bones) was observed in the knockout mice. Moreover, these mice also had a dramatically reduced ability to form trabecular bone (the “spongy” bone found near joints and in vertebrae).

Normal bone tissue undergoes continuous remodeling. Cells called osteoclasts destroy old bone, while cells called osteoblasts are involved in the formation of new bone (Figure 1). Therefore, it is possible that the loss of bone seen in the knockout mice is due to increased osteoclast activity. Indeed it is known that another type of lectin, galectin8, can increase RANKL signaling, which stimulates hematopoietic stem cells to develop into osteoclasts, hence increasing the rate at which bone is destroyed (Vinik et al., 2015). However, Yue et al. found that osteoclast numbers were not affected in knockout mice, raising the possibility that Clec11a is instead important for bone formation.

**Figure 1.**
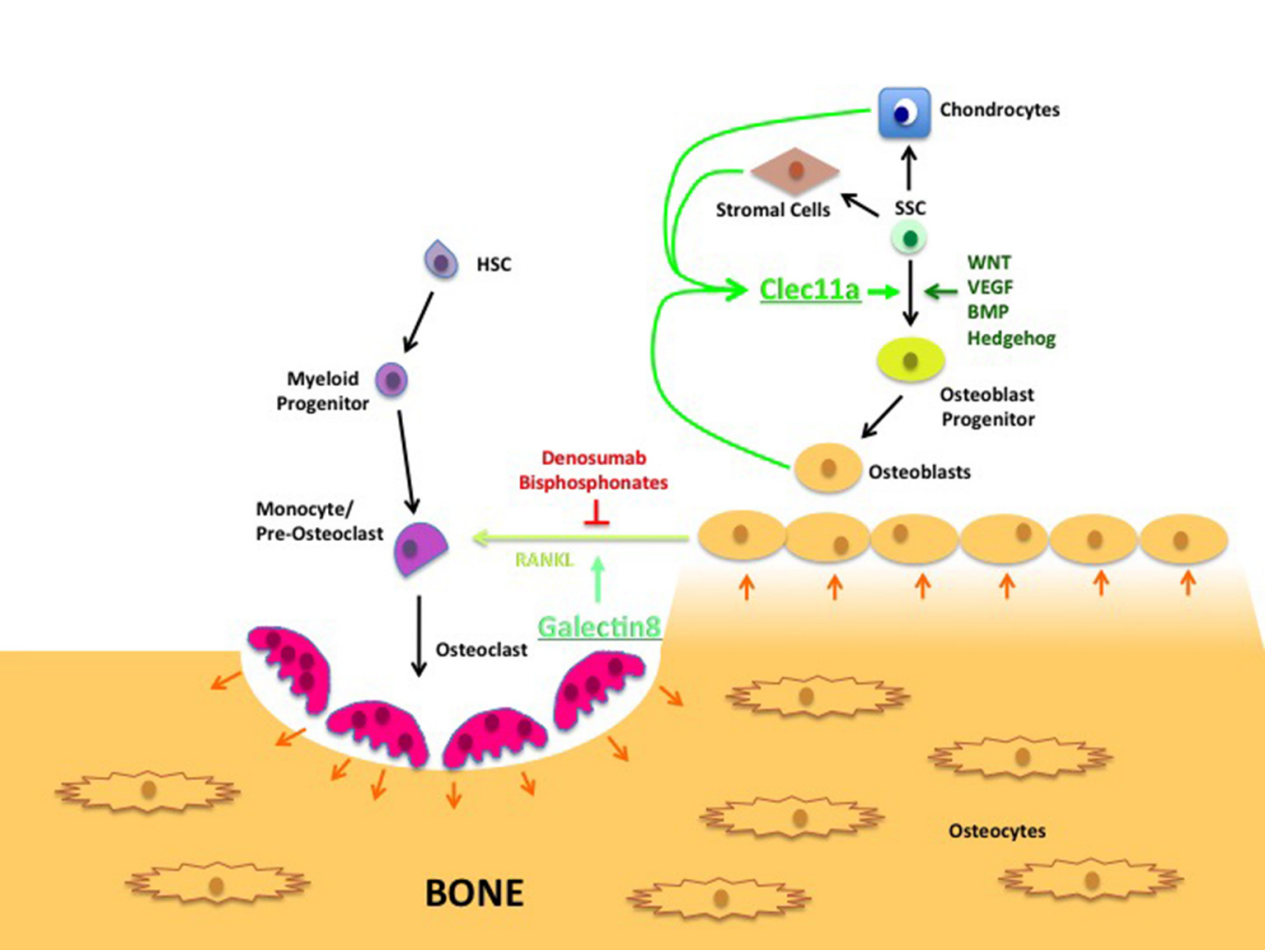
Lectin’s role in bone remodeling. In adults, bones are maintained through the destruction of old bone by cells called osteoclasts (pink), and replenished through the activity of cells called osteoblasts (yellow-brown). Hematopoietic stem cells (HSC; purple) develop through a number of intermediate cell types to become mature osteoclasts, while skeletal stem cells (SSCs; green) can develop into a number of mature cell types, including chondrocytes, stromal cells and osteoblasts. The latter produce a c-type lectin called Clec11a that stimulates the formation of osteoblast progenitors from SSCs. (The WNT, VEGF, BMP and Hedgehog signaling pathways are also involved in this process.) The osteoblasts also produce a signaling protein called RANKL that stimulates the final stage of osteoclast development; another lectin called galectin8 encourages RANKL signaling. Currently, most treatments for osteoporosis interrupt RANKL signaling using compounds such as denosumab and bisphosphonates. The results presented by Yue et al. suggest that increasing the levels of Clec11a may form part of an alternative osteoporosis treatment.

By taking advantage of recent studies that characterized skeletal stem cell lineages in mice, Yue et al. next investigated whether Clec11a deficiency affects the skeletal stem or progenitor cells that may develop into osteoblasts (Bianco and Robey, 2015; Chan et al., 2015; Marecic et al., 2015; Worthley et al., 2015; Zhou et al., 2014; Méndez-Ferrer et al., 2010; Chan et al., 2009). Using a variety of techniques including fluorescence-activated cell sorting (FACS) analysis, they found that the knockout mice had a relatively normal number of skeletal stem cells: however, these cells were much less likely to develop into osteoblasts. In vitro studies performed with a recombinant form of the Clec11a protein show that Clec11a directly promotes the formation of osteoblasts from skeletal progenitor cells. Further experiments revealed that injections of Clec11a could fully restore the bone deficiencies seen in both Clec11a knockout mice and mice that displayed the symptoms of osteoporosis. Clec11a also stimulated human skeletal progenitor cells that had been implanted into mice to form bone. Taken together, these results indicate that Clec11a is essential for maintaining the integrity of the skeleton in adults.

From a clinical perspective, these findings are especially significant given the medical burden that osteoporosis and other age-related skeletal disorders pose to the aging global population. Treatment options for these conditions are limited and typically rely on agents such as bisphosphonates and RANKL antagonists that reduce osteoclast activity. However, these treatments are plagued by significant side effects, in part because they work in a non-specific manner (Gennari et al., 2016). Clec11a seems to act specifically at the point where skeletal stem and progenitor cells commit to developing into osteoblasts, and does not appear to affect other cell lineages that develop from the skeletal stem cells. Therefore, Yue et al. have demonstrated the potential of Clec11a to form part of a precise and targeted therapy for treating bone and bone-healing deficiencies.

In future, it will be important to determine how Clec11a works mechanistically at the molecular level. Focusing on purified skeletal stem cells and the progenitor cell lineages that they develop into could reveal whether Clec11a signaling intersects with other signaling pathways that are known to be involved in the formation of the skeleton, or whether it represents an entirely new signaling mechanism (Chan et al., 2015).
